# Hydrogen Sulfide-Linked Persulfidation Maintains Protein Stability of ABSCISIC ACID-INSENSITIVE 4 and Delays Seed Germination

**DOI:** 10.3390/ijms23031389

**Published:** 2022-01-26

**Authors:** Mingjian Zhou, Jing Zhang, Heng Zhou, Didi Zhao, Tianqi Duan, Shuhan Wang, Xingxing Yuan, Yanjie Xie

**Affiliations:** 1 Laboratory Center of Life Sciences, College of Life Sciences, Nanjing Agricultural University, Nanjing 210095, China; 2020216038@stu.njau.edu.cn (M.Z.); 2018216035@njau.edu.cn (J.Z.); hengzhou@njau.edu.cn (H.Z.); 2019116100@njau.edu.cn (D.Z.); 2021116100@stu.njau.edu.cn (T.D.); 2021116101@stu.njau.edu.cn (S.W.); 2 Institute of Industrial Crops, Jiangsu Academy of Agricultural Sciences, Nanjing 210014, China; yxx@jaas.ac.cn

**Keywords:** hydrogen sulfide, persulfidation, DES1, ABI4, protein stability

## Abstract

Hydrogen sulfide (H_2_S) is an endogenous gaseous molecule that plays an important role in the plant life cycle. The multiple transcription factor ABSCISIC ACID INSENSITIVE 4 (ABI4) was precisely regulated to participate in the abscisic acid (ABA) mediated signaling cascade. However, the molecular mechanisms of how H_2_S regulates ABI4 protein level to control seed germination and seedling growth have remained elusive. In this study, we demonstrated that ABI4 controls the expression of L-CYSTEINE DESULFHYDRASE1 (DES1), a critical endogenous H_2_S-producing enzyme, and both ABI4 and DES1-produced H_2_S have inhibitory effects on seed germination. Furthermore, the ABI4 level decreased during seed germination while H_2_S triggered the enhancement of the persulfidation level of ABI4 and alleviated its degradation rate, which in turn inhibited seed germination and seedling establishment. Conversely, the mutation of ABI4 at Cys250 decreased ABI4 protein stability and facilitated seed germination. Moreover, ABI4 degradation is also regulated via the 26S proteasome pathway. Taken together, these findings suggest a molecular link between DES1 and ABI4 through the post-translational modifications of persulfidation during early seedling development.

## 1. Introduction

Growing bodies of reports have demonstrated that H_2_S, a gaseous signaling molecule, is involved in a variety of physiological processes during plant growth and development, such as autophagy, flowering, and stomatal closure [1,2,3,4]. The multiple H_2_S generation pathways have been correspondingly identified, including L/D-cysteine desulfhydrase, β-cyanoalanine synthase, and *O*-acetylserine(thiol)lyase [5,6]. Particularly, cytoplasmic L-Cys desulfhydrase DES1 was considered as one of the most critical H_2_S-producing enzymes in *Arabidopsis*. DES1 exhibited a higher affinity to L-cysteine and a strong catalytic capacity to degrade L-cysteine to sulfide, ammonia, and pyruvate [7]. In parallel, endogenous H_2_S levels reduced by 30% in the *Arabidopsis des1* mutant [7], which has brought great convenience for us to investigate the physiological effects of H_2_S in plants [8,9]. A greater understanding of the mechanism for H_2_S biological functions in plants has been achieved, accompanied by the key feature of H_2_S at protein persulfidation, one type of post-translational modification of oxidized cysteine residues to form a persulfide group (RSSH) in target proteins [10,11]. To date, persulfidated proteins in plants have been widely reported and the function of persulfation has exhibited great complexity and diversity in different tissues and organs of plants [12,13]. Persulfidation significantly impacts protein biological functions, such as enzymatic activity, subcellular localization, and protein interaction [14,15,16]. At least 5214 proteins, 13% of the entire annotated proteome in *Arabidopsis* roots, were susceptible to persulfidation [17]. However, our current understanding of persulfidation remains relatively limited compared to other protein modifications such as phosphorylation and acetylation.

Seed germination is an essential stage in the whole life cycle of plants, which is crucial for plant propagation and requires the precise coordination of multiple external and internal signals [18]. A sequence of mechanisms has been adopted to fine-tune intercellular homeostasis during seed germination in plants due to its high vulnerability to biotic and abiotic stresses [19,20]. ABSCISE ACID-INSENSITIVE 4 (ABI4), an APETALA2 (AP2)/ ETHYLENE-RESPONSIVE ELEMENT BINDING PROTEIN (EREBP) domain-containing transcription factor (TF), acts as a positive modulator in the ABA signaling pathway [21]. During seed germination and post-germination process, ABI4 is involved in regulating a wide range of important biological events, including stress response, chloroplast and mitochondria retrograde, and lipid metabolism [22,23,24,25]. ABI4 integrates with various phytohormones signaling during seed germination and flowering [23,26,27]. Interestingly, ABI4 acts as either activator or repressor to control the transcription of downstream responsive genes by recognizing coupling 1 elements (CE1) (CACCG and CCAC motif) within their promoters [21,28]. For example, ABI4 could directly control the MAPK cascade by specifically binding to the CE1 motif of the *MAPKKK18* promoter [16]. However, ABI4 inhibits the transcript abundance of the mitochondrial retrograde signal gene *ALTERNATIVE OXIDASE1a* (*AOX1a*) by targeting the CGTGAT element in the promoter [29].

Our current understandings of the mechanism of H_2_S-regulated signal transduction have been achieved in genetic and molecular studies. Our recent study has illustrated a DES1-ABI4 loop molecular regulatory mechanism to control plant responses to ABA. ABA-triggered accumulation of H_2_S leads to persulfidation of ABI4 at Cys250 [16]. Meanwhile, ABI4 binds specifically to the CE1 motif of the *DES1* promoter to control *DES1* expression and further H_2_S production. However, ABI4 transcript and protein levels are fairly low and even undetectable during vegetative growth in mature overexpressing transgenic plants [30], implying that ABI4 is precisely regulated by a complex network while exercising its function. The general mechanism behind the protein stability of ABI4 controlled by protein persulfidation and the underlying signaling pathways remain to be characterized. 

In this study, we show that ABI4 controls the expression of *DES1*, and both ABI4 and DES1-produced H_2_S have inhibitory effects on seed germination. H_2_S-linked persulfidation on ABI4 Cys250 enhances ABI4 stability, which in turn inhibits seed germination and post-germination development in *Arabidopsis*. We establish a molecular mechanism for DES1 and ABI4 synergistically in the regulation of seed germination and seedling establishment through ABI4 protein stability.

## 2. Results

### 2.1. Inhibition of Germination and Seedling Growth in Arabidopsis by Exogenous H_2_S

**Figure 1:**
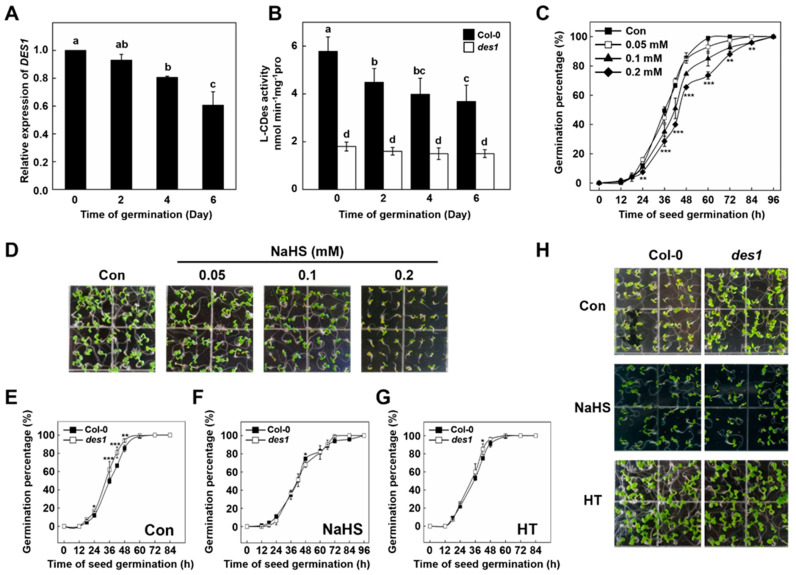
The inhibitory effect of H_2_S on the seed germination and post-germination of *Arabidopsis thaliana*. (**A**) Time-course changes of relative expression of *DES1* in wild-type (Col-0) *Arabidopsis* seedlings. *Arabidopsis* seeds were incubated on ^1^/_2_ MS medium. Afterward, the relative expression of *DES1* was determined at the indicated time points after germination. Expression levels are presented relative to that of *UBQ10*, with *DES1* expression in the 0-day set to 100%. (**B**) Time-course changes of L-CDes activity in wild-type (Col-0) and *des1* germinating seeds. L-CDes activity was determined at the indicated time points after germination. (**C**) Time-course changes of germination percentage in Col-0 *Arabidopsis* seedlings upon NaHS treatment. *Arabidopsis* seeds were incubated on ^1^/_2_ MS medium containing 0 (control), 0.05, 0.1, or 0.2 mM NaHS, respectively. The germination percentage (%) of seeds was counted at the indicated time points. Con: control. (**D**) Photographs of wild-type *Arabidopsis* seeds germinated seeds five days after the initiation of germination on ^1^/_2_ MS medium containing NaHS (0, 0.05, 0.1, 0.2 mM). (**E**–**G**) Time-course changes of germination percentage in *Arabidopsis* seedlings upon NaHS or HT treatment. (**H**) Photographs of germinated seeds of wild-type and *des1* five days after the initiation of germination on ^1^/_2_ MS medium containing NaHS (0.1 mM) or HT (1 mM). HT: hypotaurine. Data are mean ± SE of three independent experiments with three biological replicates for each individual experiment. Bars with different letters indicate significant differences at *p* < 0.05 according to one-way ANOVA (post-hoc Tukey’s HSD test). Asterisks represent significant differences between treatments according to Student’s *t*-test (* *p* < 0.05, ** *p* < 0.01, *** *p* < 0.001).

Our previous studies have revealed that persulfidation plays an indispensable role in regulating plant ABA responses, including the induction of stomatal closure [14] and the inhibition of primary root growth [16]. To determine whether H_2_S is involved in the *Arabidopsis* germination process, we measured the relative expression of *DES1* during the WT seed germination period. Quantitative PCR (qPCR) results showed that the relative expression of *DES1* decreased in a time-dependent manner from dormant seeds to 6-day-old seedlings (Figure 1A). Meanwhile, the enzyme activity of L-cysteine desulfhydrase (L-CDes) was also detected. Mutation of *DES1* resulted in a marked decrease of L-CDes activity in germinating seeds (Figure 1B). Moreover, we observed that the enzyme activity of total L-CDes decreased significantly during the progress of germination in WT but not in *des1* mutant (Figure 1B). These results further proved that l-CDes activity decrease was closely related to DES1, which may function as a negative regulator during seed germination.

To further analyze the effect of H_2_S on *Arabidopsis* seed germination, NaHS, a well-known H_2_S donor, was applied into an MS medium with different concentrations. As expected, the inhibitory effects of NaHS on *Arabidopsis* seed germination were observed over a wide range of NaHS concentrations from 0.05 to 0.2 mM (Figure 1C). These results indicated that NaHS treatment could delay the beginning of germination in *Arabidopsis* and prolong the full germination period in a concentration-dependent manner. However, NaHS-treated seeds can fully germinate at 96 h after treatment (Figure 1C). Subsequently, we examined NaHS affect the post-germination process. We observed that *Arabidopsis* cultured in different NaHS concentrations displayed growth inhibition as shown by a lower greening degree and smaller leaves of the seedlings. NaHS strengthened this inhibitory effect in a concentration-dependent manner (Figure 1D). Primary root length was measured to evaluate the effect of NaHS on post-germination growth. Accordingly, NaHS treatment inhibited primary root growth of 5-day-old *Arabidopsis* seedlings, suggesting H_2_S inhibits seedling growth (Supplementary Figure S1). Taken together, these results revealed that exogenous H_2_S delayed the initiation of seed germination and inhibited post-germination growth.

### 2.2. DES1-Produced H_2_S Inhibits Arabidopsis Seed Germination and Post-Germination Growth

To test whether DES1-produced H_2_S impacted seed germination, the germination performance of the *des1* mutant was compared with WT. Under normal conditions, *des1* mutant germinates faster compared to WT, especially 36 to 48 h after germination. However, both WT and *des1* mutant completely germinated after 72 h (Figure 1E). Subsequently, the *des1* mutant was treated with NaHS. As shown, the germination rate of both WT and *des1* displayed a similar, overall downward trend with the treatment of NaHS (Figure 1F). Long treatment with NaHS can lead to sulfide oxidation and thus form polysulfides. In this study, the treatment of chemical H_2_S donor GYY4137 resulted in a similar inhibitory effect on both WT and *des1* as NaHS, suggesting that the effects on seed germination observed are related to H_2_S directly (Supplementary Figure S2). Meanwhile, both WT and *des1* mutant seed germination was significantly accelerated by hypotaurine (HT), an H_2_S scavenger (Figure 1G).

The post-germination phenotype was also investigated. *des1* mutant displayed a faster growth trend than WT during the post-germination process (Figure 1H). The greening degree of *des1* was significantly higher than that of WT, and the leaves were larger after five days of germination under control conditions. Meanwhile, the application of NaHS inhibited the development of the leaf and delayed the greening of both *des1* and WT. By contrast, treatment of HT accelerated seedling growth and abolished the phenotypic differences between WT and *des1* (Figure 1H). Therefore, these experimental data revealed that DES1-produced H_2_S has an inhibitory effect on both germination and post-germination growth of *Arabidopsis*.

### 2.3. Functional of DES1 and ABI4 on Seed Germination and Post-Germination Growth

**Figure 2:**
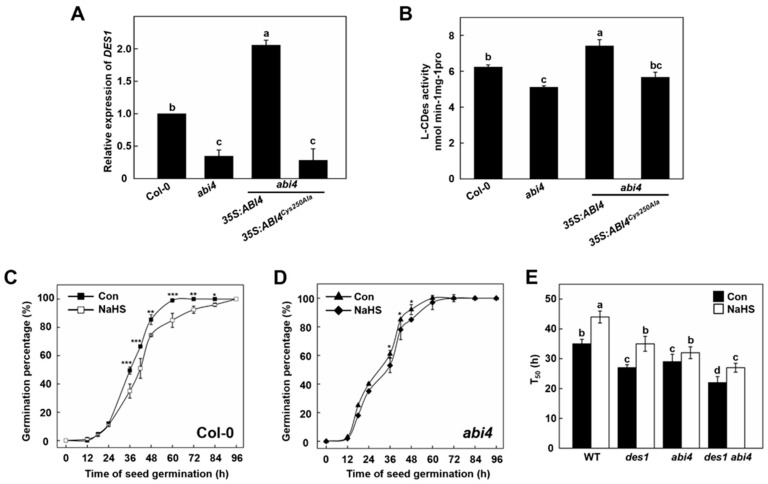
Functional analysis of DES1 and ABI4 on seed germination and post-germination growth. (**A**,**B**) Relative expression of *DES1* (**A**) and L-CDes activity (**B**) in Col-0, *abi4*, *35S:ABI4 abi4*, and *35S:ABI4^Cys250Ala^ abi4 Arabidopsis* seedlings. Five-day-old seedlings were collected for qRT-PCR analysis and measurement of L-CDes enzyme activity, respectively. Expression levels are presented relative to that of *UBQ10*, with *DES1* expression in the Col-0 set to 100%. (**C**,**D**) Time-course changes of germination percentage in Col-0 and *abi4 Arabidopsis* seedlings upon NaHS treatment. *Arabidopsis* seeds were incubated on ^1^/_2_ MS medium containing 0.1 mM NaHS, respectively. The germination percentage (%) of seeds was counted at the indicated time points. (**E**) The time to obtain 50% of germinated seeds in WT, *des1*, *abi4*, and *des1 abi4* seeds with or without 0.1 mM NaHS. Data are mean ± SE of three independent experiments with three biological replicates for each individual experiment. Bars with different letters indicate significant differences at *p* < 0.05 according to one-way ANOVA (post-hoc Tukey’s HSD test). Con: control. Asterisks represent significant differences between treatments according to Student’s *t*-test (* *p* < 0.05, ** *p* < 0.01, *** *p* < 0.001).

Our previous studies demonstrated that ABI4 can activate *DES1* transcription by binding to its promoter, and Cys250 from ABI4 is critical for its binding [16]. In this study, the qPCR analysis showed that the expression of *DES1* was significantly blocked in the 5-day-old *abi4* mutant seedling (Figure 2A). Correspondingly, *DES1* expression was higher in transgenic plants overexpressing *ABI4* but not *the ABI4^Cys250Ala^* variant (Figure 2A), implying that Cys250 is crucial for ABI4-activated *DES1* expression in *Arabidopsis* seedlings. Subsequently, the total L-CDes activity was also measured in *Arabidopsis* seedlings of WT, *abi4*, *35S:ABI4 abi4*, and *35S:ABI4^Cys250Ala^ abi4*. *abi4* and *35S:ABI4^Cys250Ala^ abi4* exhibited decreased L-CDes activities compared with WT, whereas L-CDes activity was increased in *35S:ABI4 abi4* (Figure 2B). These results demonstrated that *DES1* expression was controlled by functional ABI4 in *Arabidopsis* seedlings.

Time-course changes of germination percentage were examined in WT and *abi4 Arabidopsis* seedlings upon NaHS treatment. While the germination speed of *abi4* is faster than wild-type, NaHS-triggered inhibitory response in germination is weakened by the mutation of ABI4 (Figure 2C,D). The germination rates of *des1* and *abi4* mutant lines were further compared. Mutation of either DES1 or ABI4 reduced the time to have 50% germinated seeds (T50; Figure 2E). Meanwhile, the T50 index is significantly shortened in *des1 abi4* double mutant than its parental lines, all of which were significantly increased by NaHS treatment. Furthermore, DES1 and ABI4 also showed additive effects with regard to seedling primary root growth (Supplementary Figure S3). Taken together, these results demonstrated that H_2_S and ABI4 have additive inhibitory effects during seed germination and the post-germination stages.

### 2.4. ABI4 Protein and Its Persulfidation Level Decreases during Germination and Post-Germination Stages

**Figure 3:**
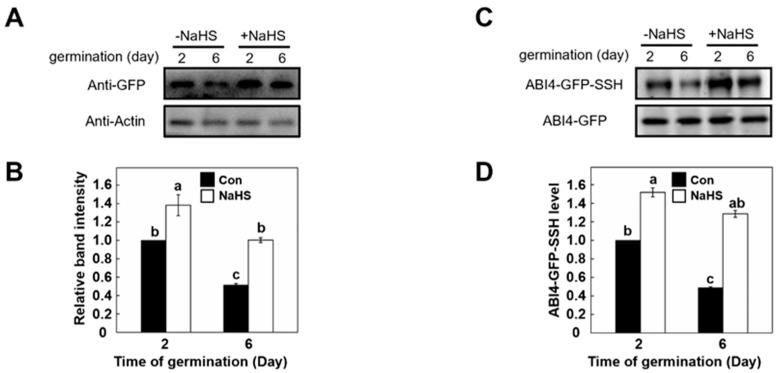
The stability and persulfidation level of ABI4 during germination and post-germination growth. (**A**) NaHS affects the ABI4 protein level In vivo. *35S:ABI4-GFP* transgenic seedlings grown on ^1^/_2_ MS medium with or without NaHS (0.1 mM) were harvested at the indicated times. Total proteins were extracted and then checked by immunoblot analysis. ABI4 proteins were detected using an anti-GFP antibody. Relative amounts of proteins were determined by densitometry normalized to actin. (**B**) Quantification of the relative band intensity shown in (**A**). (**C**) Effect of the NaHS on the persulfidation of ABI4 In vivo. Sample treatments were as described for (**A**). Total proteins were extracted and then subjected to the biotin-switch assay to analyze persulfidation levels (ABI4-GFP-SSH). Persulfidated ABI4-GFP protein was detected with anti-GFP antibody after tag-switch labeling and streptavidin purification. The bottom panels show the total ABI4-GFP used for the tag-switch assay as a loading control. (**D**) Quantification of the ABI4-GFP-SSH level is shown in (**C**). For (**B**,**D**), the bars indicate the relative abundance of the corresponding protein (**B**) or persulfidated protein (**D**) compared with that of the control un-treated sample (set to 1.0). Signals from two independent experiments were quantified. Different letters indicate significant differences at *p* < 0.05 according to one-way ANOVA (post-hoc Tukey’s HSD test).

ABI4 is a positive regulator of ABA regulation, and ABA has an inhibitory effect on the germination of *Arabidopsis* [21]. However, the expression of ABI4 was very low in vegetative growth [30]. In this study, *35S:ABI4-GFP* transgenic seedlings were used. Meanwhile, the effect of NaHS on ABI4 protein level during seed germination (2 days) and post-germination (6 days) stages were compared. The results showed that the level of ABI4 protein in the post-germination stage decreased by half compared with that of the germination stage (Figure 3A,B). However, compared to the control sample, treatment with NaHS caused a 40% increase in ABI4 protein level after two days of sampling. Meanwhile, the decreased ABI4 protein level at 6-days after germination increased by 100% after the addition of NaHS treatment. In conclusion, the ABI4 protein level decreased markedly during seed germination and post-germination growth, which can be future attenuated by the addition of H_2_S.

To verify whether the above NaHS-driven responses were related to ABI4 persulfidation, we further checked the persulfidation level of ABI4 by a biotin-switch method in the following experiment [15]. As shown in Figure 3C, the ABI4 persulfidation level of seedlings decreased by approximately 50% after six days of growth, suggesting the ABI4 presulfidation level decreased during seed germination and seedling establishment. However, the declined level of ABI4 persulfidation was reversed to a higher level in the presence of NaHS, compared with that of the 2-day sample (Figure 3D). Besides, the level of ABI4 persulfidation increased dramatically in both 2-day and 6-day samples after exposure to NaHS. These results suggested that both protein and corresponding persulfidation level of ABI4 was regulated by H_2_S. 

### 2.5. Persulfidation at Cys250 of ABI4 Inhibits Its Degradation

**Figure 4:**
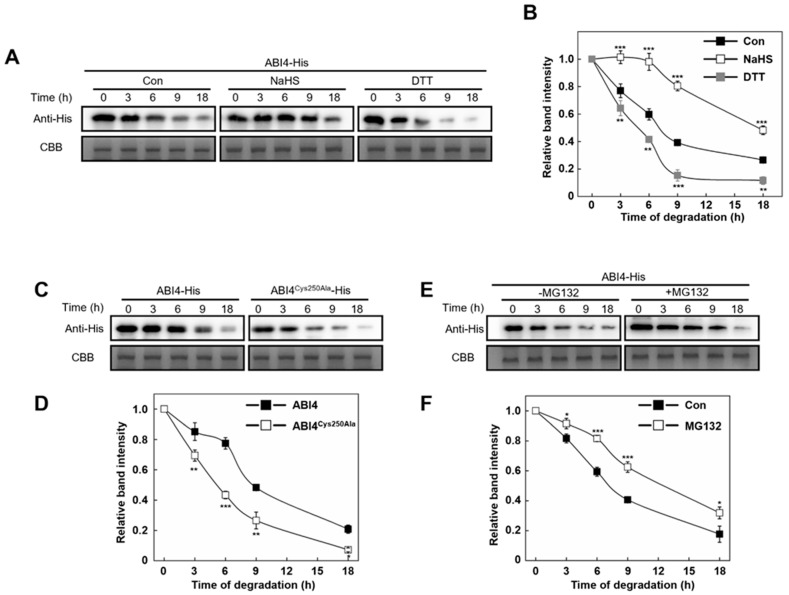
Persulfidation at Cys250 of ABI4 inhibits its degradation In vitro. (**A**) Cell-free degradation of ABI4-His recombinant protein in protein extracts from Col-0. Purified ABI4-His recombinant protein was treated with NaHS (0.1 mM) or DTT (1 mM) for 1 h, dialyzed, and then incubated with extracts of 10-day-old *Arabidopsis* seedlings at 25 °C. Protein without chemical treatment was set as control (Con). During incubation, samples were collected at indicated time points for immunoblot analysis using anti-His antibody. Samples collected before the addition of protein extracts were set as internal control, as normalized to load equal amounts determined by Coomassie brilliant blue staining (CCB). (**B**) Quantification of the relative band intensity shown in (**A**). (**C**) Cell-free degradation analysis of ABI4 mutant recombinant proteins. Purified ABI4-His and ABI4^Cys250Ala^-His recombinant protein were dialyzed and then incubated with extracts of 10-day-old *Arabidopsis* seedlings. Sample treatments and measurements were as described for (**A**). (**D**) Quantification of the relative band intensity shown in (**C**). (**E**) The effect of MG132 on the stability of ABI4 in vitro. Purified ABI4-His recombinant proteins were dialyzed and then incubated with extracts containing 50 µm MG132 of 10-day-old *Arabidopsis* seedlings. Sample treatments and measurements were as described for (**A**). (**F**) Quantification of the relative band intensity shown in (**E**). For the quantification of relative band intensity, the data indicate the relative abundance of the corresponding protein compared with that of the control sample (set to 1.0). Signals from two independent experiments were quantified. Asterisks represent significant differences between treatments according to Student’s *t*-test (* *p* < 0.05, ** *p* < 0.01, *** *p* < 0.001).

We hypothesized that persulfidation of ABI4 may regulate its protein level during germination. To test whether persulfidation affects ABI4 stability, we set up a cell-free assay using ABI4-His recombinant protein purified from *Escherichia coli*, treating with either NaHS or dithiothreitol (DTT) followed by dialysis. The level of ABI4-His recombinant protein was detected after 3–18 h incubation with total protein extract from WT, followed by using immunoblotting with anti-His antibody. With an increased incubation period, the ABI4-His protein level was gradually decreased, which was further attenuated or exacerbated by NaHS or DTT treatment, respectively (Figure 4A,B). For example, the ABI4-His protein level decreased by 60% after 9 h of incubation, while this value is 20% or 85% after treatment of NaHS or DTT, respectively. Moreover, the decreased tendency of ABI4-His protein level became faster when its Cys250 was mutated to Ala (Figure 4C,D), which showed a maximum decrease of 40% at 6 h of incubation. Meanwhile, treatment of NaHS or DTT enhance or decrease the persulfidation level of recombinant ABI4 protein, whereas Cys250Ala mutation almost fully abolished the persulfidation of recombinant ABI4 protein (Supplementary Figure S4). These results further indicated persulfidation on Cys250 is critical for ABI4 stability.

To further investigate whether the decrease of ABI4 level is also attributed to the ubiquitin-26S proteasome pathway, MG132, a specific 26S proteasome inhibitor of ubiquitin-mediated protein degradation, was used. In this study, ABI1 protein, an important component of ABA signaling, where its degradation was regulated by ubiquitination [31,32], was used as a positive control. In vitro and In vivo experiments demonstrated that MG132 sufficiently blocks ABI1 degradation (Supplementary Figure S5). As expected, the decrease level of ABI4-His recombinant protein was relieved by the application MG132 to some extent, with maximum mitigation effect at 6 h by 25% (Figure 4E,F). These results indicated that the ABI4 Cys250 persulfidation regulates its degradation In vitro, and this process is partially related to 26S proteasome activity.

### 2.6. The Stability of ABI4 Is Regulated by Its Persulfidation

**Figure 5:**
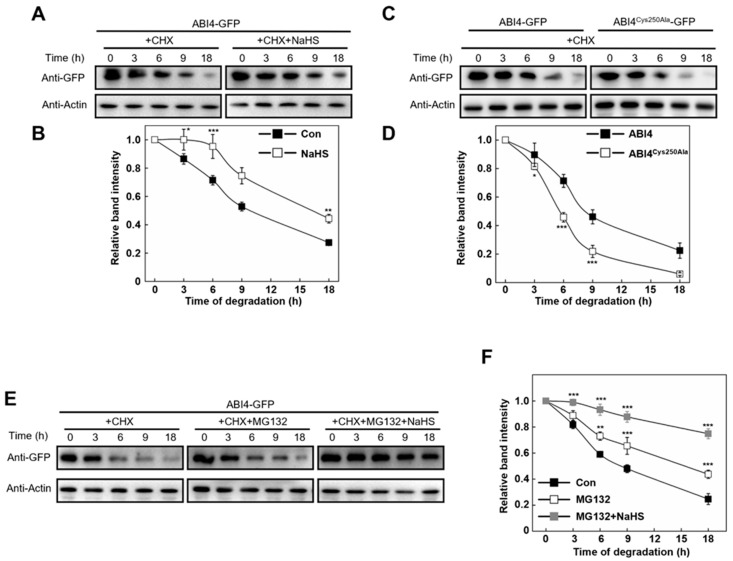
The stability of ABI4 is regulated by its persulfidation In vivo. (**A**) The effect of NaHS on the stability of ABI4 In vivo. *Arabidopsis* protoplasts isolated from the Col-0 lines were transfected with ABI4-GFP expressing plasmid constructs. After incubation in low light for 12 h, protoplasts were treated with 0.1 mM NaHS (or distilled water, as a control) and 150 μM CHX (to block protein translation) for 1 h. Then protoplasts were lysed and incubated at 30 °C after being divided into five tubes. Samples were stopped at indicated time points and checked by immunoblot analysis. Proteins were detected using an anti-GFP antibody, and relative amounts of proteins were determined by densitometry normalized to actin. (**B**) Quantification of the relative band intensity shown in (**A**). (**C**) The stability of wild-type ABI4 or its Cys250Ala mutated version In vivo. *Arabidopsis* protoplasts isolated from the wild-type lines were transfected with ABI4-GFP or ABI4^Cys250Ala^-GFP expressing plasmid constructs, respectively. Sample harvest and measurements were as described for (**A**). (**D**) Quantification of the relative band intensity shown in (**C**). (**E**) The effect of MG132 and NaHS on the stability of ABI4 In vivo. Protoplasts were treated with 50 µm MG132 or 50 µm MG132 and 0.1 mM NaHS, respectively (or distilled water, as a control) for 1 h, and other sample treatments and measurements were as described for (**A**). (**F**) Quantification of the relative band intensity shown in (**E**). For the quantification of relative band intensity, the data indicate the relative abundance of the corresponding protein compared with that of the control sample (set to 1.0). Signals from two independent experiments were quantified. Asterisks represent significant differences between treatments according to Student’s *t*-test (* *p* < 0.05, ** *p* < 0.01, *** *p* < 0.001).

To investigate the effect of persulfidation on protein stability of ABI4 In vivo, a protoplast-based time course degradation assay was performed. The protoplasts from WT plants were transfected with equal amounts of plasmids expressing either ABI4-GFP or ABI4^Cys250Ala^-GFP, respectively. The protoplasts expressing ABI4-GFP were treated with or without NaHS before lysed. Cycloheximide (CHX) was used to inhibit protein synthesis during protein extraction. As shown in Figure 5A,B, NaHS treatment attenuated the decrease of ABI4-GFP level during the whole detection period compared with the control sample, indicating that NaHS enhanced the stability of ABI4-GFP In vivo. However, the possibility that NaHS might inhibit proteasome activity and thus indirectly enhance the stability of ABI4 cannot be ruled out. 

Cys250 mutation almost completely abolished ABI4 persulfidation (Supplementary Figure S4). Next, the degradation rate between ABI4-GFP and ABI4^Cys250Ala^-GFP were compared. Notably, our results exhibited that the ABI4^Cys250Ala^-GFP mutant version caused a decrease rate which increased to approximately 80% at 9 h, whereas ABI4-GFP only decreased by 50% (Figure 5C,D). These results demonstrated that Cys250 residue is necessary for maintaining the ABI4-GFP level. Subsequently, the following experiment demonstrated that MG132 attenuated the decreased tendency of the ABI4-GFP level in a time-dependent manner (Figure 5E,F), indicating the involvement of the 26S proteasome-mediated degradation pathway. The persulifdation level of ABI4-GFP was increased by NaHS, regardless of the presence of MG132 (Supplementary Figure S6). Furthermore, the degradation rate of ABI4-GFP was further inhibited by the application of NaHS in the presence of MG132, especially during 0–3 h of treatment. After 9 h of incubation, the degradation rate was inhibited by 40% with the treatment of both MG132 and NaHS (Figure 5E,F). Taken together, these results indicated that persulfidation regulates ABI4 protein stability, in which the pathway of 26S proteasome may involve.

## 3. Discussion

In this study, we focused on the effects of H_2_S and ABI4 on seed germination and post-germination growth under normal physiological conditions. While ABA levels decreased gradually during seed germination, we found that ABI4 and DES1-produced H_2_S have an inhibitory effect on seed germination synergistically. We established a molecular framework for H_2_S-linked persulfidation maintaining protein stability of ABI4 in the regulation of *Arabidopsis* seed germination and post-germination process. During this process, ABI4 protein was degraded concurrently and its persulfidation level decreased, which was closely related to H_2_S/DES1. ABI4 controls the expression of *DES1,* and both ABI4 and DES1-produced H_2_S have inhibitory effects during seed germination and post-germination growth. We further discovered persulfidation regulates ABI4 stability during seed germination and post-germination growth in *Arabidopsis*.

Multiple lines of evidence obtained from genetic and physiological studies demonstrate that DES1-produced H_2_S has an inhibitory effect on both germination and post-germination growth of *Arabidopsis*. This idea is supported by the observation that seed germination and post-germination development was inhibited by NaHS in a concentration-dependent manner (Figure 1D). Consistently, the *des1* mutant showed faster germination compared with WT. Our data further suggested that ABI4 regulates *DES1* expression, as *DES1* expression and L-CDes activities in *abi4* mutants was severely reduced from that in the WT, respectively. These results may be supported by the fact that ABI4 activates *DES1* transcription by binding to its promoter [16]. Interestingly, the germination of WT, *des1*, *abi4*, and *des1 abi4* seeds were all significantly inhibited by the treatment of NaHS (Figure 2E). Based on these findings, this study provides new evidence for the notion that ABI4 integrates with DES1 as a negative regulator to participate in seed germination coordinately.

ABI4 plays an essential role as one of the positive regulation transcription factors mediating ABA-dependent stress response [25]. The ABI4 protein level is precisely regulated by a complex network under a vegetative state [30]. In this study, protein degradation analysis revealed that MG132, a specific 26S proteasome, relieved the decrease level of ABI4 protein, indicating that ABI4 degradation was also regulated via the 26S proteasome pathway, which was also found in ABI1 and ABI5 [32,33]. However, a recent study has found that phytoplasma SAP05 (secreted AY-WB proteins) mediates the concurrent degradation of SPL and GATA developmental regulators via hijacking the plant ubiquitin receptor RPN10 independent of substrate ubiquitination [34]. Thus, the degradation of ABI4 may also not be dependent on ubiquitination entirely. Interestingly, the accumulation of ABI4 in seeds was observed at the early stage of germination, and its steady-state mRNA levels dropped sharply a few days after germination [35]. Importantly, the ABI4 level in the NaHS treated seedlings was evidently higher than in the control. These results further implied that H_2_S might also be involved in the mechanism of regulation of ABI4 stability to control seed germination.

H_2_S is emerging as a potential messenger molecule involved in the modulation of various aspects of physiological processes in plants [36,37]. Signaling by H_2_S is proposed to occur via persulfidation, a post-translational modification of protein Cys residues (R-SHs) to form persulfides (R-SSH). Our results revealed that the ABI4 presulfidation level decreased during seed germination (Figure 3C), and the ABI4 protein stability could be attenuated or accelerated by NaHS or DTT (Figure 4A). Furthermore, mutation of Cys250 to Ala accelerated ABI4 decrease tendency (Figure 4C and Figure 5C). Collectively, these results illustrated that the persulfidation of ABI4 was also linked to ABI4 stability.

In conclusion, we discovered that H_2_S could greatly trigger the enhancement of the persulfidation level of ABI4 and alleviate its degradation rate, which in turn inhibits *Arabidopsis* germination. The discovery of ABI4 protein stability regulated by its persulfidation expands our understanding of H_2_S role in plant signal transduction networks and establishes a molecular framework for the crosstalk between different post-translational modifications during seed germination.

## 4. Materials and Methods

### 4.1. Plant Materials and Growth Conditions

*Arabidopsis* mutants of *des1* (SALK_103855; Col-0) and *abi4* (SALK_080095; Col-0) mutants were obtained from the Arabidopsis Biological Resource Center (http://www.arabidopsis.org/abrc, accessed on 23 March 2020). Cross *abi4* with *des1* to obtain the double mutant *des1 abi4*. *35S:ABI4-GFP* transgenic materials were obtained from Dr. Wei Chi (Institute of Botany, The Chinese Academy of Sciences, Beijing, China). Seeds were disinfected with sodium hypochlorite for 20 min and then washed three times with sterile water. They were cultured in Petri dishes on semi-solid Murashige and Skoog (^1^/_2_ MS) medium (pH 5.8). The plants were grown in a 16 h/8 h (23 °C/18 °C) growth chamber using a bulb-type fluorescent lamp with a light intensity of 100 mmol photons m^−2^ s^−1^ irradiation.

### 4.2. Molecular Cloning

#### 4.2.1. For Expression in *Escherichia coli*

The fragment of ABI4 amplified by PCR was introduced into the PET28a vector (for His fusion) using a homologous recombination technique (Vazyme, Nanjing, China) with the enzyme digestion sites *NdeI* and *XhoI*. Site-directed mutagenesis was performed using a Mut Express II Fast Mutagenesis Kit (Vazyme). All procedures followed the manufacturer’s manual. The specific primers used for *ABI4* are listed in Supplementary Data 1.

#### 4.2.2. For Transient Expression in *Arabidopsis* Protoplasts

Homologous recombination technology (Vazyme) was used to transfer PCR amplified fragments into the PAN580 vector at *XbaI* and *BamHI*. *Arabidopsis* protoplasts were extracted according to the method previously described by Yoo et al. [38] with some modifications. The constructed transient expression vector PAN580 was transformed into *Arabidopsis* protoplasts combined with PEG calcium-mediated method and cultured in the dark at 22 °C for 16 h. The protoplasts expressing target GFP were identified by fluorescence microscopy and used for transient expression analysis.

#### 4.2.3. For Expression in Planta

ABI4 fragments were cloned into the pCAMBIA 1302 vector. The constructed plasmid was transferred into a competent cell of *Agrobacterium tumefaciens* and then transformed into *Arabidopsis* using the inflorescence infection method. ^1^/_2_ MS medium with 50 mg/mL hygromycin B was used to select transgenic plants. PCR, fluorescence observation, and western blot analysis were combined to identify transgenic plants.

### 4.3. Real-Time RT-PCR Analysis

Mature *Arabidopsis* seedlings were collected for RNA extraction. According to the manufacturer’s instructions, seedlings were ground using a mortar and pestle in liquid nitrogen until a fine powder appeared, and then separated total RNA using RNA-easy Isolation Reagent (Vazyme, Nanjing, China). 1000 ng RNA from seedlings was used to synthesize the first-strand cDNA in a 20 μL reaction volume (Vazyme, Nanjing, China) using 1 μM of primers. According to the manufacturer’s instructions, the AceQ qPCR SYBR green master mix (Vazyme) was used for real-time RT-PCR in the Mastercycler^®^ep realplex real-time PCR system (Eppendorf, Germany). The specific primers used are listed in Supplementary Data 1.

### 4.4. Determination of Activity of L-Cysteine Desulfhydrase

The method for determining L-cysteine desulfhydrase activity was described by Riemenschneider et al. [39] with some modifications. 0.2 g of seedlings was collected and ground with liquid nitrogen and the soluble protein was extracted using 1 mL 20 mM pH 8.0 Tris-HCl. The protein concentration was determined using a BCA kit (Takara, Dalian, China) and calibrated to be consistent. The release of H_2_S was determined to evaluate L-cysteine desulfhydrase activity. 100 mM Tris HCl (pH 9.0), 2.5 mM DTT, 0.8 mM L-cysteine and 10 µg protein solutions were mixed to 1 mL. The reaction was initiated by the addition of L-cysteine and terminated by 100 µL 30 mM FeCl_3_ and 100 µL 20 mM N, N-dimethyl-ρ-phenylenediamine dihydrochloride after incubation at 37 °C for 15 min in the dark. The content of H_2_S was measured by colorimetric at 670 nm. Taking the known Na_2_S concentration as the standard curve, the activity of L-cysteine desulfhydrase was expressed as nmol g^−1^ FW min^−1^.

### 4.5. Seed Germination and Green Open Cotyledon Assays

The seeds were germinated and grown on ^1^/_2_ MS medium with or without NaHS, if there were no other instructions in the text. The percentage of germinated seeds and green cotyledons were recorded, and the seedlings were photographed at the designated time points.

### 4.6. Expression and Purification of Recombinant Protein

The recombinant protein was expressed and purified in BL21 competent cells (Vazyme). 0.1 mM IPTG was added, and the bacteria were grown to OD600 = 0.4 to 0.6 at 16 °C for 12 h. After enriching the bacterial solution, suspend the pellet in PBS buffer and use an ultrasonic breaker. The protein was broken and centrifuged at 12,000× *g* for 30 min, and then the extract was collected for purification. NI-NTA pre-packaged gravity column (Sangon Biotech, Shanghai, China) was used to purify the His-labeled protein, and the protein purification procedure was performed in accordance with the column specifications.

### 4.7. SDS-PAGE and Immunoblotting

Protein extracts were separated by 12.5% SDS-PAGE. The electrophoresis was ended when the bromophenol blue was moved to 5 mm below the gel, and the gel was transferred to a polyvinylidene fluoride membrane (Roche, Shanghai, China) for 60 min at 100 V on ice using a wet transfer method. The membrane was rinsed with deionized water, then immersed in a blocking buffer (5% skim milk), placed on a decolorizing shaker, and incubated slowly for one hour or overnight at 4 °C. Place the membrane in TBST with a sufficient amount of primary antibody and incubate at room temperature for 2 h with gentle shaking. After the primary antibody incubation, the membrane was washed three times with TBST for 7 min each time. The appropriate HRP-labeled secondary antibody was labeled according to the source of the primary antibody, it was diluted in the corresponding proportion, and shaken gently at room temperature for 1 h. The horseradish peroxidase HRP-ECL luminescence method was used to perform the immunoblot analysis. The software ImageJ (https://imagej.nih.gov/ij/, accessed on 31 December 2021) was used to quantify protein abundance, and signals from two independent experiments were quantified. Full-sized membrane scans are presented in Supplemental Figure S7.

### 4.8. Immunochemical Detection of S-Persulfidated Proteins

The protein persulfidation level was detected with a tag-switch method described by Aroca et al. [15] with modifications. *35S:ABI4-GFP* transgenic seedlings were grown in ^1^/_2_ MS medium. Seedling extracts were dissolved with protein extraction buffer (25 mM pH8.0 Tris-HCl, 100 mM NaCl, and 0.2% Triton X-100) containing protease inhibitor (Yeasen, Shanghai, China). The extract was centrifuged at 4 °C for 10 min and centrifuged at 12,000× *g* for 10 min. Take 80 μL supernatant and add 320 μL blocking buffer (50 mM methylsulfonylbenzothiazole was dissolved in tetrahydrofuran), incubated at 37 °C for 1 h to block free sulfhydryl groups. The protein was precipitated twice with acetone, the pellet was resuspended with buffer (50 mM pH 8.0 Tris-HCl, 2.5% SDS, and 20 mM HPDP-biotin), and then incubated at 37 °C in the dark for 3 to 4 h. 33 μL of protein loading buffer was added without β-mercaptoethanol. It was then incubated at 95 °C for 5 min, the protein was separated with 12.5% SDS-PAGE, transferred to the PVDF membrane, and the level of persulfidation was detected with an anti-GFP antibody (Beyotime, Shanghai, China). 

### 4.9. Cell-Free Protein Degradation Assay

The cell-free protein degradation assay was performed as described with some modifications [32]. 10-day-old WT seedlings were harvested, homogenized in liquid nitrogen, and suspended in degradation buffer (25 mM pH 7.4 Tris-HCl, 10 mM MgCl_2_, 50 mM NaCl, 1 mM DTT, 0.2% Triton X-100, 5 mM ATP, and 1 mM PMSF). The lysis was centrifuged at 12,000× *g* at 4 °C for 10 min. 100 ng purified recombinant ABI4-His protein was incubated with 500 μg total proteins in a total volume of 100 μL for each reaction. Samples were kept at 25 °C. The reactions were stopped at indicated times by adding a 5× SDS loading buffer. The samples were incubated at 95 °C for 5 min and subjected to western blot analysis with anti-His antibody (Beyotime, Shanghai, China).

### 4.10. Protoplast-Based Protein Degradation Assay

Protoplasts were lysed in 200 µL degradation buffer (25 mM pH 7.4 Tris-HCl, 50 mM NaCl, 1 mM DTT, 1 mM PMSF, 5 mM ATP and 0.2% Triton X-100) with 150 µM CHX. The lysis was divided into 5 tubes (80 µL each) and incubated at 30 °C. The reaction was terminated by adding 20 µL 5 × SDS loading buffer at the indicated time points. Samples were kept on ice until all the reactions were completed, then incubated at 95 °C for 5 min and subjected to Western blot analysis with anti-GFP antibody (Beyotime, Shanghai, China).

### 4.11. Statistical Analysis

The statistical analysis and graph construction were performed using SPSS v16.0 (https://www.ibm.com/products/spss-statistics, accessed on 30 December 2021). Differences were considered significant at *p* < 0.001, 0.01, or 0.05.

## Data Availability

Not applicable.

## Supplementary Materials

The following are available online at https://www.mdpi.com/article/10.3390/ijms23031389/s1.

## References

[1] Frederick D.D., Nair S.P., Ward P.D. (2013). Increased growth and germination success in plants following hydrogen sulfide administration. PLoS ONE.

[2] Laureano-Marín A.M., Aroca Á., Pérez-Pérez M.E., Yruela I., Jurado-Flores A., Moreno I., Gotor C. (2020). Abscisic acid-triggered persulfidation of the cysteine protease ATG4 mediates regulation of autophagy by sulfide. Plant Cell.

[3] Zhang H., Hu S.L., Zhang Z.J., Hu L.Y., Jiang C.X., Wei Z.J., Liu J., Wang H.L., Jiang S.T. (2011). Hydrogen sulfide acts as a regulator of flower senescence in plants. Postharvest Biol. Technol..

[4] Scuffi D., Álvarez C., Laspina N., Gotor C., Lamattina L., García-Mata C. (2014). Hydrogen sulfide generated by L-cysteine desulfhydrase acts upstream of nitric oxide to modulate ABA-dependent stomatal closure. Plant Physiol..

[5] Arenas-Alfonseca L., Gotor C., Romero L.C., Garcıá I. (2018). ß-cyanoalanine synthase action in root hair elongationis exerted at early steps of the root hair elongation pathway and is independent of direct cyanide inactivation of NADPH oxidase. Plant Cell Physiol..

[6] Zhang J., Zhou M.J., Zhou H., Zhao D.D., Gotor C., Romero L.C., Shen J., Ge Z.L., Zhang Z.R., Shen W.B. (2021). Hydrogen sulfide (H_2_S), a signaling molecule in plant stress responses. J. Integr. Plant Biol..

[7] Álvarez C., Calo L., Romero L.C., Garcia I., Gotor C. (2010). An O-acetylserine(thiol)lyase homolog with L-cysteine desulfhydrase activity regulates cysteine homeostasis in *Arabidopsis*. Plant Physiol..

[8] Jin Z.P., Xue S.W., Luo Y.N., Tian B.H., Fang H.H., Li H., Pei Y.X. (2013). Hydrogen sulfide interacting with abscisic acid in stomatal regulation responses to drought stress in *Arabidopsis*. Plant Physiol. Biochem..

[9] Zhang J., Zhou M.J., Ge Z.L., Shen J., Zhou C., Gotor C., Romero L.C., Duan X.L., Liu X., Wu D.L. (2020). ABA-triggered guard cell L-cysteine desulfhydrase function and in situ H_2_S production contributes to heme oxygenase-modulated stomatal closure. Plant Cell Environ..

[10] Aroca A., Gotor C., Romero L.C. (2018). Hydrogen sulfide signaling in plants: Emerging roles of protein persulfidation. Front. Plant Sci..

[11] Moseler A., Dhalleine T., Rouhier N., Couturier J. (2021). *Arabidopsis thaliana* 3-mercaptopyruvate sulfurtransferases interact with and are protected by reducing systems. J. Biol. Chem..

[12] Arif Y., Hayat S., Yusuf M., Bajguz A. (2021). Hydrogen sulfide: A versatile gaseous molecule in plants. Plant Physiol. Biochem..

[13] Filipovic M.R., Jovanović V.M. (2017). More than just an intermediate: Hydrogen sulfide signalling in plants. J. Exp. Bot..

[14] Shen J., Zhang J., Zhou M.J., Zhou H., Cui B.M., Gotor C., Romero L.C., Fu L., Yang J., Foyer C.H. (2020). Persulfidation-based modification of cysteine desulfhydrase and the NADPH oxidase RBOHD controls guard cell abscise acid signaling. Plant Cell.

[15] Aroca A., Benito J.M., Gotor C., Romero L.C. (2017). Persulfidation proteome reveals the regulation of protein function by hydrogen sulfide in diverse biological processes in *Arabidopsis*. J. Exp. Bot..

[16] Zhou M.J., Zhang J., Shen J., Zhou H., Zhao D.D., Gotor C., Romero L.C., Fu L., Li Z.M., Yang J. (2021). Hydrogen sulfide-linked persulfidation of ABSCISIC INSENSITIVE 4 controls *Arabidopsis* ABA responses through the transactivation of mitogen-activated protein kinase kinase kinase 18. Mol. Plant.

[17] Jurado-Flores A., Romero L.C., Gotor C. (2021). Label-free quantitative proteomic analysis of nitrogen starvation in *Arabidopsis* root reveals new aspects of H_2_S signaling by protein persulfidation. Antioxidants.

[18] Weitbrecht K., Müller K., Leubner-Metzger G. (2011). First off the mark: Early seed germination. J. Exp. Bot..

[19] Nambara E., Okamoto M., Tatematsu K., Yano R., Seo M., Kamiya Y. (2010). Abscisic acid and the control of seed dormancy and germination. Seed Sci. Res..

[20] Zhou M.J., Zhou H., Shen J., Zhang Z.R., Gotor C., Romero L.C., Yuan X.X., Xie Y.J. (2021). H_2_S action in plant life cycle. Plant Growth Regul..

[21] Chandrasekaran U., Luo X.F., Zhou W.G., Shu K. (2020). Multifaceted signaling networks mediated by Abscisic Acid Insensitive 4. Plant Commun..

[22] Shu K., Zhang H.W., Wang S.F., Chen M.L., Wu Y.R., Tang S.Y., Liu C.Y., Feng Y.Q., Cao X.F., Xie Q. (2013). ABI4 regulates primary seed dormancy by regulating the biogenesis of abscisic acid and gibberellins in *Arabidopsis*. PLoS Genet..

[23] Shu K., Chen Q., Wu Y.R., Liu R.J., Zhang H.W., Wang P.F., Li Y.L., Wang S.F., Tang S.Y., Liu C.Y. (2016). ABI4 mediates antagonistic effects of abscisic acid and gibberellins at transcript and protein levels. Plant J..

[24] Nott A., Jung H.S., Koussevitzky S., Chory J. (2006). Plastid-to-nucleus retrograde signaling. Annu. Rev. Plant Biol..

[25] Wind J.J., Peviani A., Snel B., Hanson J., Smeekens S.C. (2012). ABI4: Versatile activator and repressor. Trends Plant Sci..

[26] Huang X., Zhang X., Gong Z., Yang S., Shi Y. (2017). ABI4 represses the expression of type-A ARRs to inhibit seed germination in *Arabidopsis*. Plant J..

[27] Aroca A., Gotor C., Bassham D.C., Romero L.C. (2020). Hydrogen sulfide: From a toxic molecule to a key molecule of cell life. Antioxidants.

[28] Luo X.F., Dai Y.J., Zheng C., Yang Y.Z., Chen W., Wang Q.C., Chandrasekaran U., Du J.B., Liu W.G., Shu K. (2021). The ABI4-RbohD/VTC2 regulatory module promotes reactive oxygen species (ROS) accumulation to decrease seed germination under salinity stress. New Phytol..

[29] Giraud E., Van Aken O., Ho L.H.M., Whelan J. (2009). The transcription factor ABI4 is a regulator of mitochondrial retrograde expression of ALTERNATIVE OXIDASE1a. Plant Physiol..

[30] Shkolnik-Inbar D., Bar-Zvi D. (2011). Expression of ABSCISIC ACID INSENSITIVE 4 (ABI4) in developing *Arabidopsis* seedlings. Plant Signal. Behav..

[31] Pan W.B., Lin B.Y., Yang X.Y., Liu L.J., Xia R., Li J.G., Wu Y.R., Xie Q. (2020). The UBC27-AIRP3 ubiquitination complex modulates ABA signaling by promoting the degradation of ABI1 in *Arabidopsis*. Proc. Natl. Acad. Sci. USA.

[32] Kong L.Y., Cheng J.K., Zhu Y.J., Ding Y.L., Meng J.J., Chen Z.Z., Xie Q., Guo Y., Li J.G., Yang S.H. (2015). Degradation of the ABA co-receptor ABI1 by PUB12/13 U-box E3 ligases. Nat. Commun..

[33] Albertos P., Romero-Puertas M.C., Tatematsu K., Mateos I., Sánchez-Vicente I., Nambara E., Lorenzo O. (2015). S-nitrosylation triggers ABI5 degradation to promote seed germination and seedling growth. Nat. Commun..

[34] Huang W.J., MacLean A.M., Sugio A., Kuo C.H., Kuo R.G.H., Hogenhout S.A. (2021). Parasitic modulation of host development by ubiquitin-independent protein degradation. Cell.

[35] Söderman E.M., Brocard I.M., Lynch T.J., Finkelstein R.R. (2000). Regulation and function of the *Arabidopsis* ABA-insensitive 4 gene in seed and abscisic acid response signaling networks. Plant Physiol..

[36] Chen S.S., Jia H.L., Wang X.F., Shi C., Wang X., Ma P.Q., Wang J., Ren M.J., Li J.S. (2020). Hydrogen sulfide positively regulates abscisic acid signaling through persulfidation of SnRK2.6 in Guard Cells. Mol. Plant.

[37] Chen S.S., Wang X.F., Jia H.L., Li F.L., Ma Y., Liesche J., Liao M.Z., Ding X.T., Liu C.X., Chen Y. (2021). Persulfidation-induced structural change in SnRK2.6 establishes intramolecular interaction between phosphorylation and persulfidation. Mol. Plant.

[38] Yoo S.D., Cho Y.H., Sheen J. (2007). *Arabidopsis* mesophyll protoplasts: A versatile cell system for transient gene expression analysis. Nat. Protoc..

[39] Riemenschneider A., Nikiforova V., Hoefgen R., De Kok L.J., Papenbrock J. (2005). Impact of elevated H_2_S on metabolite levels, activity of enzymes and expression of genes involved in cysteine metabolism. Plant Physiol. Biochem..

