# Supplementary material for: Hydrogen Sulfide-Linked Persulfidation Maintains Protein Stability of ABSCISIC ACID-INSENSITIVE 4 and Delays Seed Germination
Source: Int J Mol Sci. 2022 Jan 26;23(3):1389. doi: 10.3390/ijms23031389 (PMC8835735; doi:10.3390/ijms23031389)
Supplement: Supplementary file 1 [file ijms-23-01389-s001.zip › 20220103 Figure S.pptx]

## Slide 1
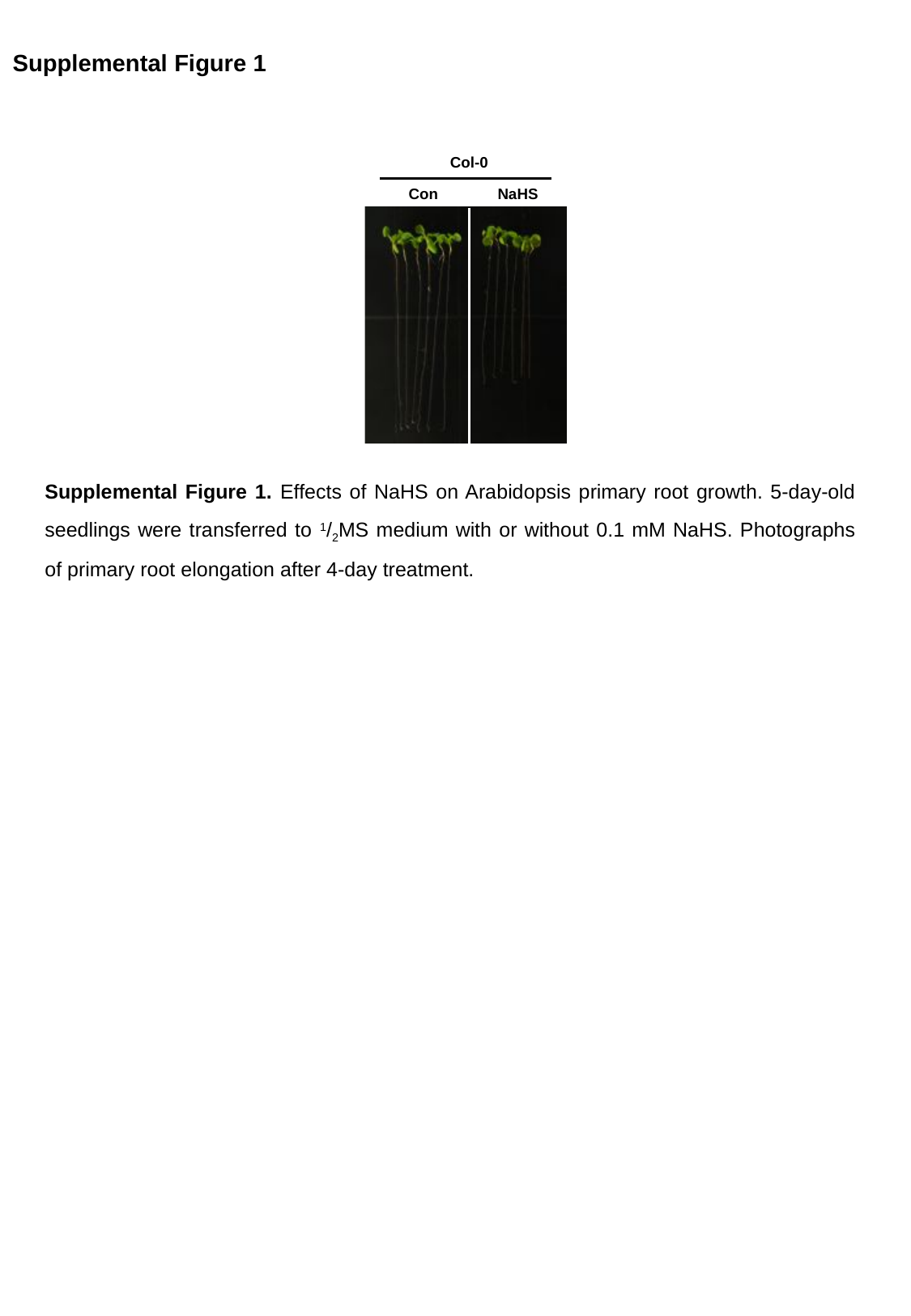

Supplemental Figure 1
Col-0
Con
NaHS
Supplemental Figure 1. Effects of NaHS on Arabidopsis primary root growth. 5-day-old seedlings were transferred to 1/2MS medium with or without 0.1 mM NaHS. Photographs of primary root elongation after 4-day treatment.

## Slide 2
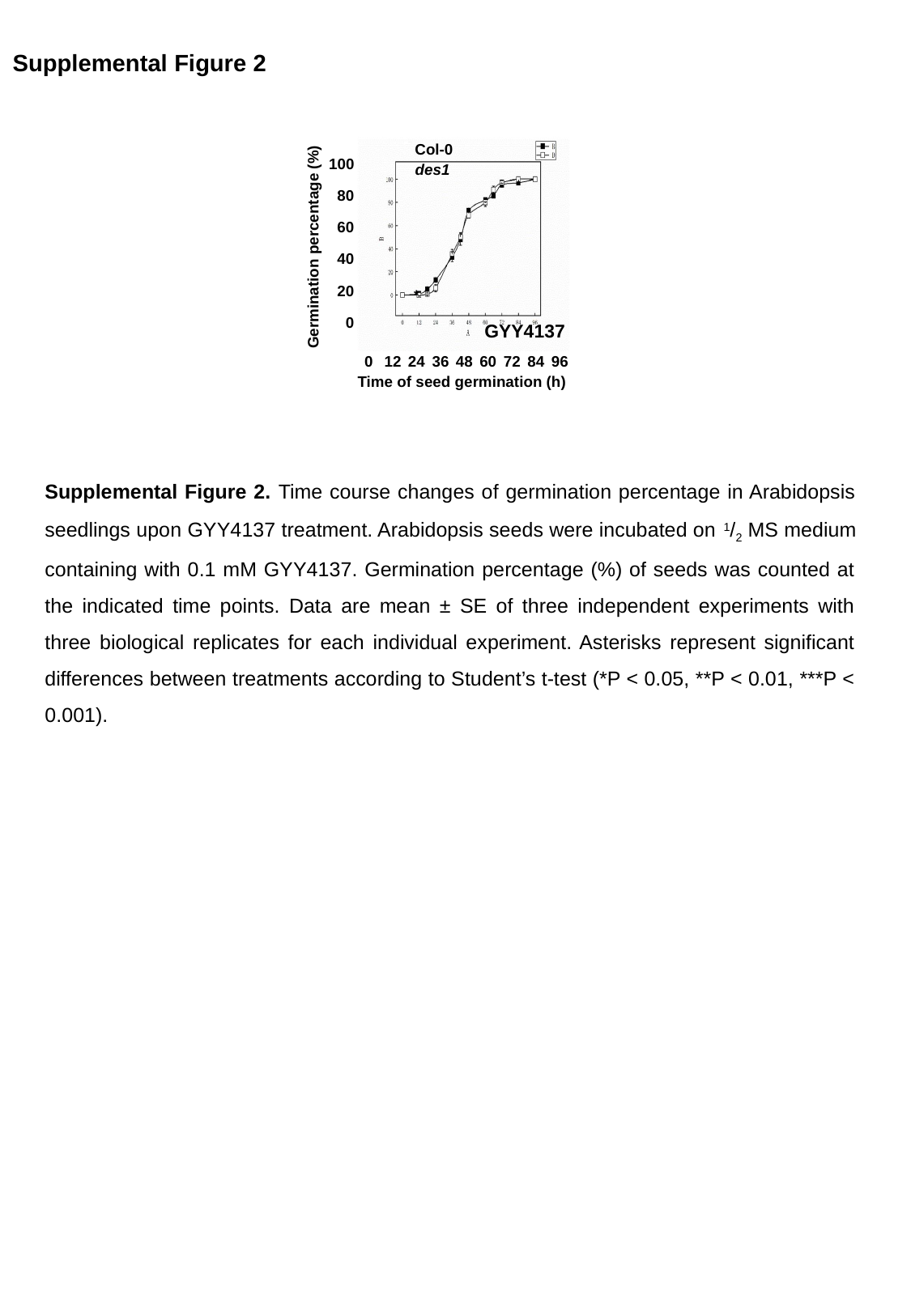

Supplemental Figure 2
Col-0
des1
100
80
60
Germination percentage (%)
40
20
*
0
GYY4137
0
12
24
36
48
60
72
84
96
Time of seed germination (h)
Supplemental Figure 2. Time course changes of germination percentage in Arabidopsis seedlings upon GYY4137 treatment. Arabidopsis seeds were incubated on 1/2 MS medium containing with 0.1 mM GYY4137. Germination percentage (%) of seeds was counted at the indicated time points. Data are mean ± SE of three independent experiments with three biological replicates for each individual experiment. Asterisks represent significant differences between treatments according to Student’s t-test (*P < 0.05, **P < 0.01, ***P < 0.001).

## Slide 3
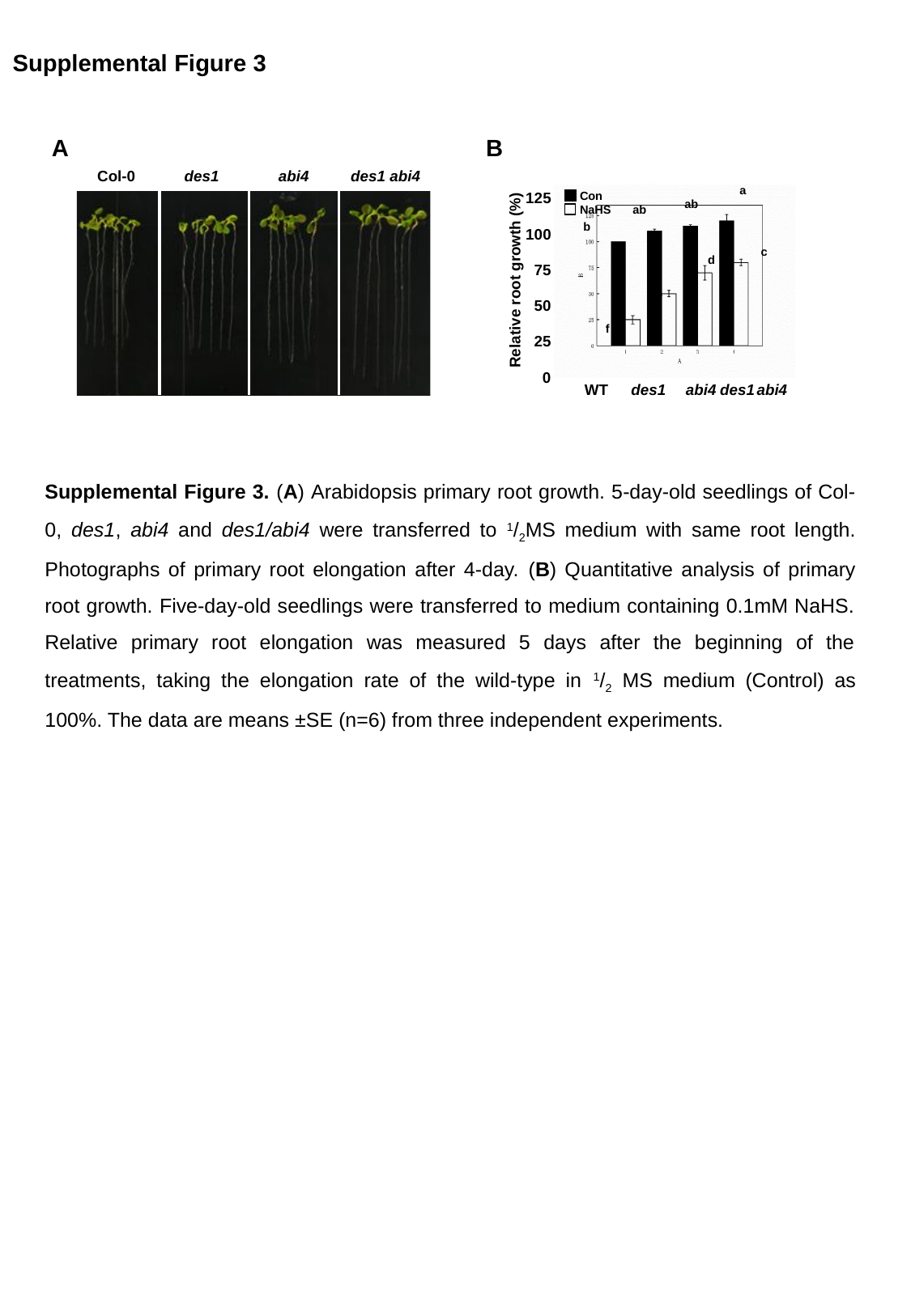

Supplemental Figure 3
A
B
Col-0
des1
abi4
des1 abi4
a
Con
NaHS
125
ab
ab
b
100
c
d
75
Relative root growth (%)
e
50
f
25
0
WT
des1
abi4
des1 abi4
Supplemental Figure 3. (A) Arabidopsis primary root growth. 5-day-old seedlings of Col-0, des1, abi4 and des1/abi4 were transferred to 1/2MS medium with same root length. Photographs of primary root elongation after 4-day. (B) Quantitative analysis of primary root growth. Five-day-old seedlings were transferred to medium containing 0.1mM NaHS. Relative primary root elongation was measured 5 days after the beginning of the treatments, taking the elongation rate of the wild-type in 1/2 MS medium (Control) as 100%. The data are means ±SE (n=6) from three independent experiments.

## Slide 4
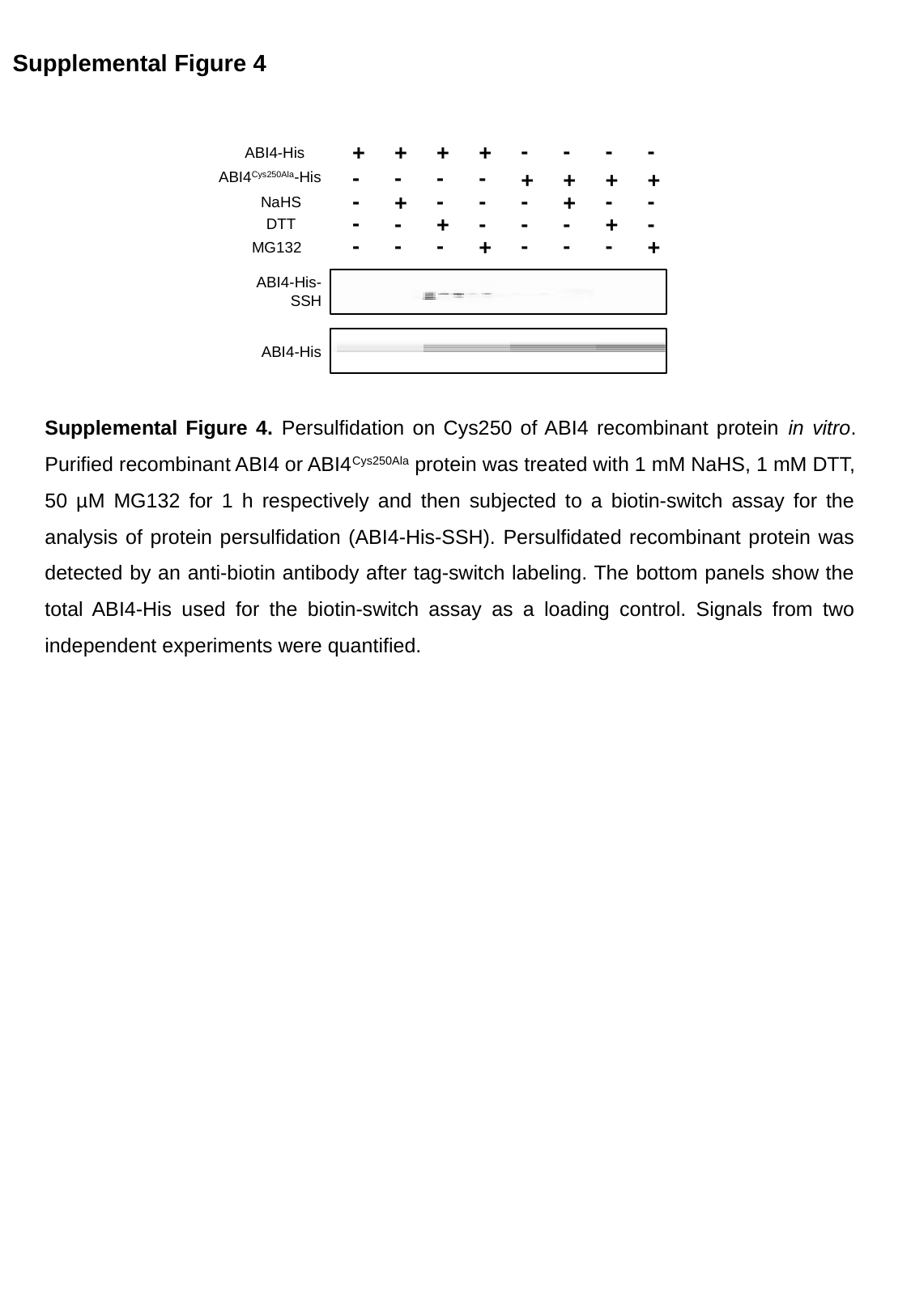

Supplemental Figure 4
-
-
-
-
+
+
+
+
ABI4-His
-
-
-
-
+
+
+
+
ABI4Cys250Ala-His
-
-
-
-
-
-
+
+
NaHS
-
-
-
-
-
-
+
+
DTT
-
-
-
-
-
-
+
+
MG132
ABI4-His-SSH
ABI4-His
Supplemental Figure 4. Persulfidation on Cys250 of ABI4 recombinant protein in vitro. Purified recombinant ABI4 or ABI4Cys250Ala protein was treated with 1 mM NaHS, 1 mM DTT, 50 µM MG132 for 1 h respectively and then subjected to a biotin-switch assay for the analysis of protein persulfidation (ABI4-His-SSH). Persulfidated recombinant protein was detected by an anti-biotin antibody after tag-switch labeling. The bottom panels show the total ABI4-His used for the biotin-switch assay as a loading control. Signals from two independent experiments were quantified.

## Slide 5
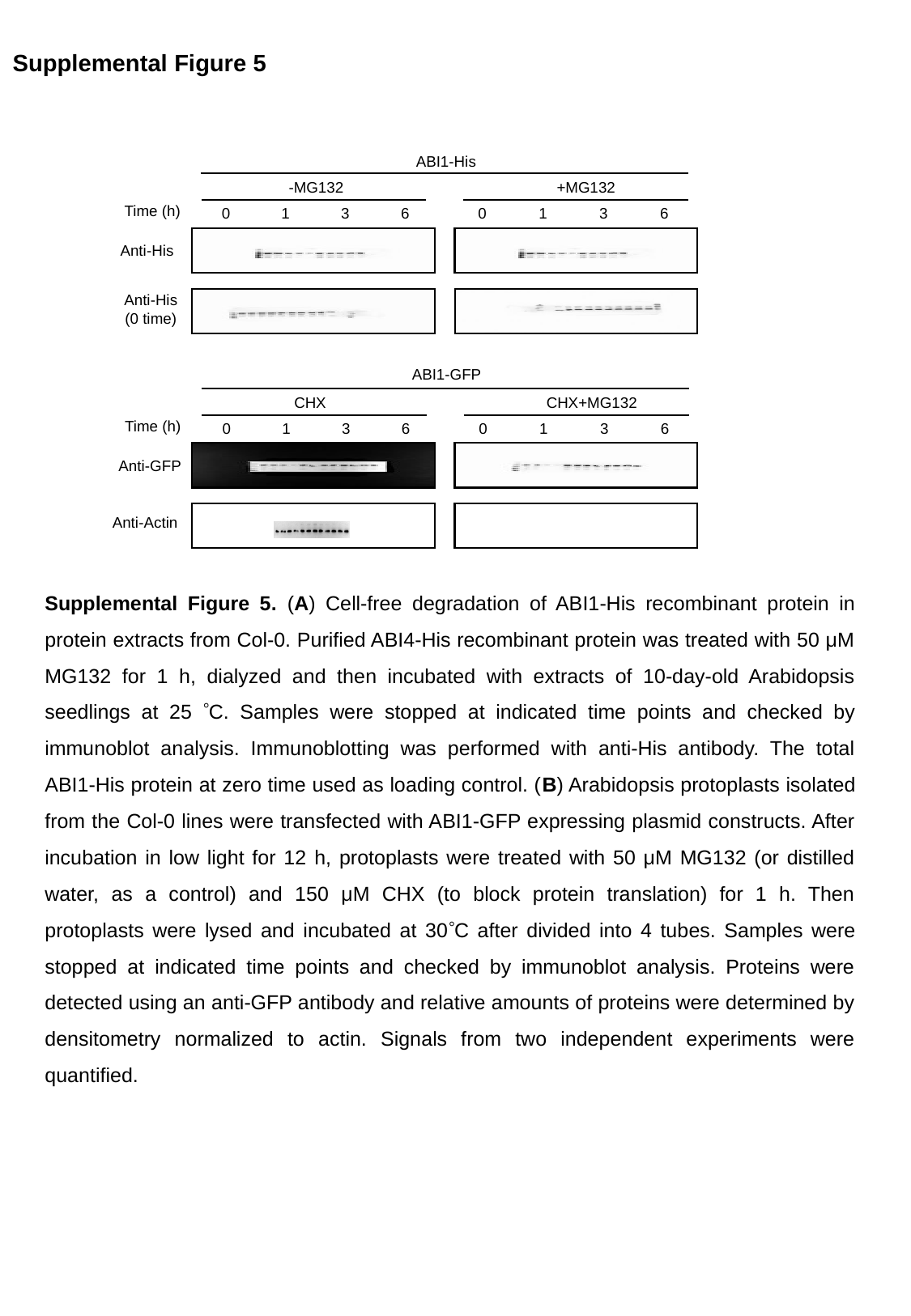

Supplemental Figure 5
ABI1-His
-MG132
+MG132
Time (h)
0
1
3
6
0
1
3
6
Anti-His
Anti-His (0 time)
ABI1-GFP
CHX
CHX+MG132
Time (h)
0
1
3
6
0
1
3
6
Anti-GFP
Anti-Actin
Supplemental Figure 5. (A) Cell-free degradation of ABI1-His recombinant protein in protein extracts from Col-0. Purified ABI4-His recombinant protein was treated with 50 μM MG132 for 1 h, dialyzed and then incubated with extracts of 10-day-old Arabidopsis seedlings at 25 °C. Samples were stopped at indicated time points and checked by immunoblot analysis. Immunoblotting was performed with anti-His antibody. The total ABI1-His protein at zero time used as loading control. (B) Arabidopsis protoplasts isolated from the Col-0 lines were transfected with ABI1-GFP expressing plasmid constructs. After incubation in low light for 12 h, protoplasts were treated with 50 μM MG132 (or distilled water, as a control) and 150 μM CHX (to block protein translation) for 1 h. Then protoplasts were lysed and incubated at 30°C after divided into 4 tubes. Samples were stopped at indicated time points and checked by immunoblot analysis. Proteins were detected using an anti-GFP antibody and relative amounts of proteins were determined by densitometry normalized to actin. Signals from two independent experiments were quantified.

## Slide 6
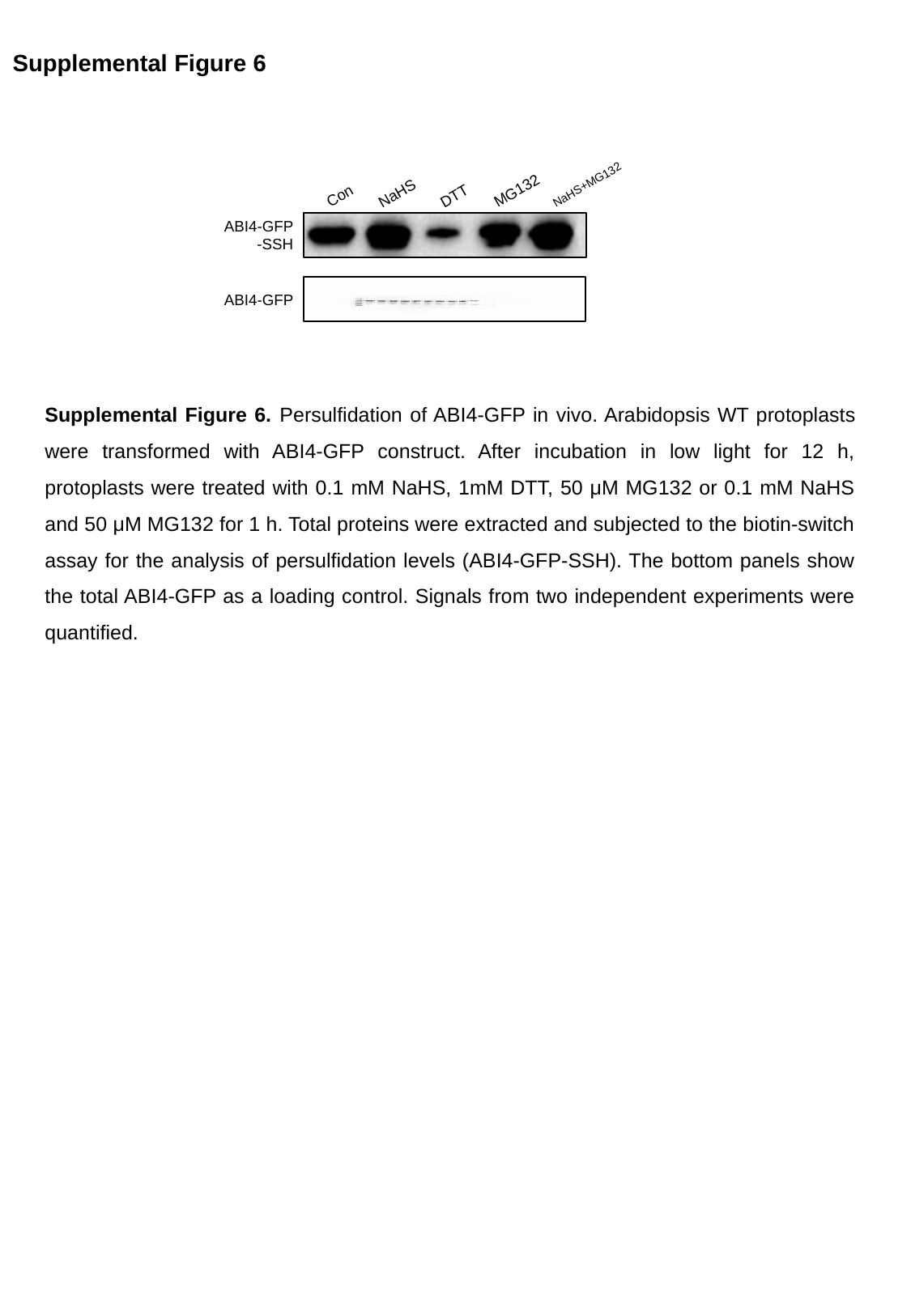

Supplemental Figure 6
NaHS+MG132
MG132
NaHS
Con
DTT
ABI4-GFP
-SSH
ABI4-GFP
Supplemental Figure 6. Persulfidation of ABI4-GFP in vivo. Arabidopsis WT protoplasts were transformed with ABI4-GFP construct. After incubation in low light for 12 h, protoplasts were treated with 0.1 mM NaHS, 1mM DTT, 50 μM MG132 or 0.1 mM NaHS and 50 μM MG132 for 1 h. Total proteins were extracted and subjected to the biotin-switch assay for the analysis of persulfidation levels (ABI4-GFP-SSH). The bottom panels show the total ABI4-GFP as a loading control. Signals from two independent experiments were quantified.

## Slide 7
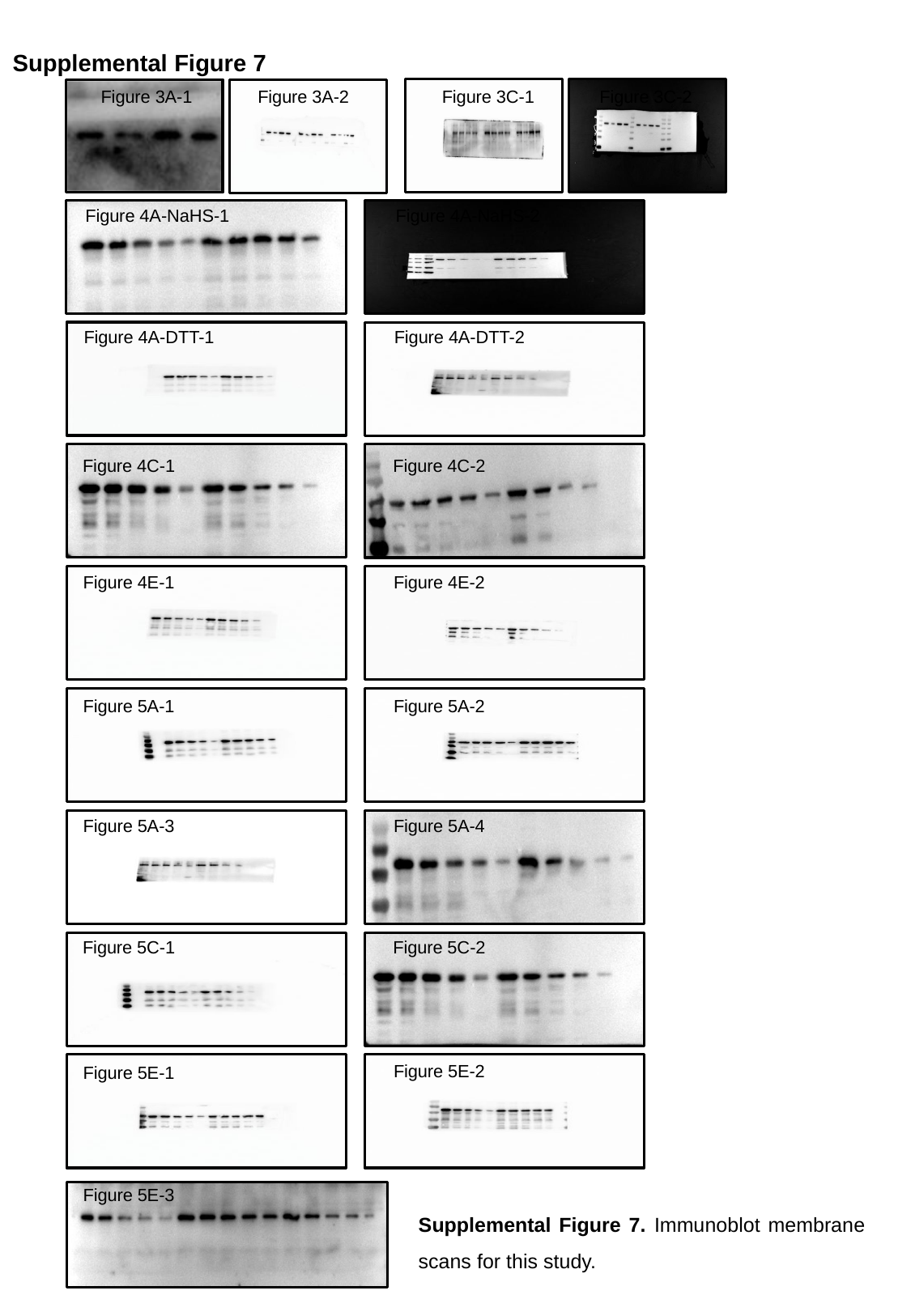

Supplemental Figure 7
Figure 3A-1
Figure 3A-2
Figure 3C-1
Figure 3C-2
Figure 4A-NaHS-1
Figure 4A-NaHS-2
Figure 4A-DTT-1
Figure 4A-DTT-2
Figure 4C-1
Figure 4C-2
Figure 4E-1
Figure 4E-2
Figure 5A-1
Figure 5A-2
Figure 5A-3
Figure 5A-4
Figure 5C-1
Figure 5C-2
Figure 5E-2
Figure 5E-1
Figure 5E-3
Supplemental Figure 7. Immunoblot membrane scans for this study.
